# Suggestions To Evaluate Whether T-wave Alternans Is T-wave Amplitude Dependent

**Published:** 2009-03-15

**Authors:** John E Madias

**Affiliations:** Mount Sinai School of Medicine of the New York University and the Division of Cardiology, Elmhurst Hospital Center, New York, NY

**Keywords:** T-wave Alternans, T-wave Amplitude

T-wave alternans (TWA) has been employed in the selection of patients considered for cardioverter/defibrillator (ICD) implantation, and has been found to have an excellent negative predictive value for sudden death and malignant ventricular arrhythmias in patients with a variety of cardiac pathologies [1]. Although a qualitative approach in the characterization of patients with TWA positive or negative results has prevailed, based on a threshold of ≥1.9 μV, attained during exercise stress testing, employing the frequency domain analysis [2], some have advanced the argument that employment of TWA in quantitative terms may have advantages  [3,4].  Indeed even the currently employed qualitative (yes or no) spectral analysis method with the ≥1.9 μV threshold value has a quantitative underpinning, in the sense that non attainment of the threshold value of ≥1.9 μV renders the patient's test negative [2]. Intuitively the magnitude of TWA must be of importance not only because a particular threshold needs to be reached, before the patient is considered positive, but because pathophysiological derangements (e.g., ischemia, volume overload, or myocardial necrosis) result in an increase of the magnitude of TWA or an association of the magnitude of the TWA and the severity of derangement  [5-7], while therapeutic interventions (e.g. beta-blockers) lead to a decrease in the magnitude of TWA  [8].

It has been speculated that the magnitude of the TWA may be T-wave amplitude dependent [9], and thus it might be of value to adjust the measured TWA values by the amplitudes (or other attributes) of the corresponding T-waves [10]. The resulting TWA index, it was further theorized, might contribute to the reproducibility of TWA in serially  assessed testing, by correcting the measured changed values by the altered amplitudes of the corresponding T-waves, which do not necessarily imply changes in the degree of the arrhythmic or sudden death risk [11]. Also normalizing TWA to T-wave amplitude may improve the specificity and positive predictive power of TWA for the arrhythmic and sudden death risk An additional objective for pursuing an investigation to improve the sensitivity and specificity of TWA for predicting sudden death is that the present form of the test, and the criteria for establishing a positive or negative test, yields an indeterminate result in a relatively high proportion of patients (20-40% by some estimates). A large number of patients with cardiomyopathy are on beta-blockers, and this is often the reason why such patients cannot reach target heart rates, and consequently their TWA tests are rendered indeterminate. In addition to inducing an indeterminate test, beta-blockers lead to an attenuated TWA value, even in patients who manage to mount a target heart rate for the elicitation of TWA [8]. Is it possible that normalization of TWA to the T-wave amplitude could reduce the number of indeterminate results? A hint that there may be an association between the magnitude of TWA and the amplitude of the T-waves (independent of the risk of sudden death and malignant arrhythmias) is that patients with intraventricular conduction abnormalities, regardless of the pattern, tend to have higher values for TWA, and a lower test specificity, i.e., higher rate of false positive results  [12-14], that probably can be attributed to the larger secondary T-waves seen in association with intraventricular conduction abnormalities [15]. The analysis proposed by the author will be important in the interpretation of negative vs. positive TWA test results in patients with cardiomyopathy and varying degrees of intraventricular conduction delay where the T-wave amplitude may be a confounder. This issue could be evaluated by correlating the measured values of the TWA in the different conventional ECG leads or the vector magnitude lead used for the calculation of the TWA, and the amplitudes of the corresponding T-waves. This author has tried to engage various authors in doing this by commenting on their articles in various journals, in letters to the Editor. However evaluating the dependence of TWA on the amplitude of the T-waves by comparing such relationship in ECGs with normal as opposed to abnormal intraventricular conduction, or in serial ECGs in the same patients are not the ideal settings for investigating this matter due to the inherent confounding factors present: the conduction problem in the former, and the different time points (with possible change in the interim of arrhythmic risk) in the latter. Assessing the relationship between TWA magnitude values in μV and T-wave attributes, e.g. amplitude in mV or μV could be done within the constraints of a single TWA test, by correlating the TWA values and T-wave amplitudes in the different conventional ECG leads, orthogonal X, Y, Z leads, and the vector magnitude lead. Mere inspection of a TWA spectral analysis report reveals that the TWA and T-wave amplitude in all the above ECG leads differ in value, and logical consequence of such realization is whether there is any relationship between these variables. The normalization of the TWA by the amplitude of the corresponding T-wave may be crude and should probably constitute the first step in this process. Other T-wave attributes could be used for normalization, such as the area under the associated T-wave, the T-wave duration, averaging the amplitude of the first and second halves of the T-wave, or any other mathematical treatment of the J-T interval is used in the calculation of the TWA by either the time-domain or the frequency-domain analyses.

 In a recent article, Paz et al [16] reported on a software program they developed and tested in 25 patients with ICDs, with simultaneous TWA assessment from the ICD electrograms (EGM) and the surface conventional ECGs, during incremental pacing. Also these authors analyzed stored ICD ECGs for TWA prior to the emergence of ventricular arrhythmias, and they found an 84% concordance rate between TWA via the EGM and the surface ECG, 85% sensitivity and 84% specificity, by setting threshold TWA values for positivity at ≥30 μV for EGMs and at the conventional ≥1.9 μV for the surface ECGs. They concluded that values in μV for EGMs were at least 10 times larger than the ones in surface ECGs, and that these 2 different ECG modalities measure the same phenomenon. Christini et al, in an accompanied editorial [17] commented on the previous experiences in assessing TWA via EGMs in animal models and the importance of closing the loop by employing this method of TWA in patients with ICDs. Here again there is an opportunity to evaluate the relationship of the magnitude of TWA with the amplitude of the corresponding T-waves, although the confounding factors of the difference in the nature of leads (EGMs vs. surface ECGs) and the fact that Paz et al used signal processing to maximize the amplitude of the T-waves in the EGMs [16,17], should be acknowledged. Another important issue in this study was that the magnitude of the TWA was largest in the EGMs from the electrode configuration using the right ventricular (distal) coil and the ICD housing (can) [16,17]. The authors used various EGM electrode configurations in the patients with the ICDs (Ring-Can, RVtip-Ring, Coil-Can); in patients with implanted cardiac resynchronization systems they used all the above and some additional EGM electrode configurations (LVtip-Can, and LVtip-RVtip). Table 2 of their article [16] lists the TWA values, calculated by the 10 different electrode configurations for EGMs and pacing modes, which they vary  from 51±38 μV  to 138±91 μV. Now this experience provides a marvelous opportunity to correlate the magnitude of the TWA with the T-wave amplitude in the corresponding T-waves. Such correlations could be carried out with EGMs with unaltered T-waves, or after employing dynamic range adjustment (signal processing maximizing the T-wave amplitude) to achieve large T-waves, as the authors did [16], provided that treatment of the T-waves would be the same for all EGM electrode configurations. It is logical to ascribe the variation of the TWA values to one or more attributes of the T-waves (e.g., amplitude) resulting from the difference in the EMG electrode configuration. There is support from the literature regarding this speculation; DeCaprio et al have compared bipolar and unipolar EGMs in patients with permanent pacemakers and found that although the amplitude of the QRS complexes were very similar in the RVtip-Can (unipolar) and RVtip-Ring (bipolar) electrode configurations, the amplitude of the T-waves in the former was larger than in the latter, by a mean of 28% [18]. Thus it is fortunate that the issue whether or not the TWA magnitude is T-wave amplitude-dependent can be resolved using an existing dataset, with some additional analyses of the TWA and T-waves.

Even if the suggestion to evaluate whether T wave alternans is T wave amplitude dependent is implemented, and leads to a TWA index with improved performance, we will still be left with a major subset of patient population in whom risk stratification for sudden cardiac death cannot be done, because mainly the results of the TWA assessment had been indeterminate. Reducing the percentage of indeterminate results would be a major scientific advancement. However one cannot predict whether sensitivity and specificity of TWA would increase by taking into account the T-wave attributes, until this hypothesis is tested. It is possible that some of the indeterminate results are secondary to these T-wave attributes, and thus adjustment for such amplitudes may enhance the chances of getting a determinate result. However there is no proof for such hypothesis either. Certainly many patients end up with a TWA indeterminate result for the plain reason that their ECG comprised excessive arrhythmic activity, or they cannot attain during exercise the necessary rise of their heart rate required for TWA assessment. Finally one cannot foresee what would be the impact of metabolic abnormalities, e.g., hyperkalemia, or cardiac memory, due to pacing, transient episodes of intraventricular blocks, or ventricular tachycardia, on a TWA index.
